# Sedentary Behavior Is Independently Related to Fat Mass among Children and Adolescents in South China

**DOI:** 10.3390/nu8110667

**Published:** 2016-10-25

**Authors:** Hongmei Xue, Guo Tian, Ruonan Duan, Liming Quan, Li Zhao, Min Yang, Lars Libuda, Rebecca Muckelbauer, Guo Cheng

**Affiliations:** 1 Department of Nutrition, Food Safety and Toxicology, West China School of Public Health, Sichuan University, Chengdu 610041, China; xhmei_1109@163.com (H.X.); tg_723@126.com (G.T.); 2 Department of Clinical Nutrition, Jincheng People’s Hospital, Jincheng 048026, China; duanrn121@163.com; 3 Office of Scientific Research Management, West China School of Public Health, Sichuan University, Chengdu 610041, China; quanlm2002@126.com; 4 West China Research Center for Rural Health Development, Sichuan University Huaxi Medical Center, Chengdu 610041, China; zhaoli@scu.edu.cn (L.Z.); yangmin2013@scu.edu.cn (M.Y.); 5 Department of Child and Adolescent Psychiatry, University Hospital Essen, University of Duisburg-Essen, Essen, Duisburg 47057, Germany; Lars.Libuda@uni-due.de; 6 Department of Pediatrics, Research Institute for the Prevention of Allergies and Respiratory Diseases in Childhood, Marien-Hospital Wesel, Wesel 46483, Germany; 7 Berlin School of Public Health, Charité—Universitätsmedizin, Berlin 10117, Germany; rebecca.muckelbauer@charite.de

**Keywords:** sedentary time, physical activity, body fat, energy intake

## Abstract

We aim to explore the independent associations of sedentary behaviors (SB) with body mass distribution among Chinese children. Data on the screen-based sedentary time (television viewing and computer use) and doing homework, physical activities and dietary intake of 1586 Chinese children (50.3% girls) aged 7–15 years were obtained through validated questionnaires. Skin-fold thickness, body height, and weight were measured to calculate percent body fat (%BF), fat mass index (FMI), and fat-free mass index (FFMI). Parental characteristics were collected by questionnaires. Among girls, time of SB (screen time or doing homework) was positively related to %BF, FMI, and FFMI (*p* < 0.03) after adjusting for maternal overweight, the average annual income of family, moderate-to-vigorous physical activity energy expenditure, and energy intake: Girls in the highest tertile of screen time/homework had 16.7%/23.3% higher relative FMI and 2.9%/2.9% higher relative FFMI than girls in the lowest tertile. Among boys, screen time was positively associated with FFMI (*p* < 0.003), but not related to %BF and FMI (*p* > 0.09), while time of doing homework was positively related to %BF and FMI (*p* = 0.03). Sedentary behaviors might be positively and independently related to fat mass among Chinese children, and were more pronounced in girls.

## 1. Introduction

The increasing prevalence of childhood obesity constitutes one of the most important threats to public health in China [1]. Although the specific causes of childhood obesity are complex, a sustained positive energy balance is linked with weight gain [2]. Sedentary behaviors (SB), e.g., screen-based SB (television viewing and computer games), might contribute to this trend. To date, observational studies on this issue among Western children [3,4,5,6,7,8] yield controversial conclusions: one cross-sectional study [3] and one prospective study [4] showed that time spent on self-reported television viewing was directly associated with body fat mass in U.S. children aged 5–11 years [4] and in British children aged 2–6 years [3], independent of physical activity (PA). Other prospective studies suggested that the relationship between SB and body fat mass was not independent of PA among children [5,6,7,8]. However, energy intake (EI), an important potential factor for energy balance, has not yet been considered among these studies.

Current international recommendations for children suggest that screen-based SB such as television viewing and computer/video games should be limited to 2 h/day [9,10,11,12]. However, the proportion of Chinese children and adolescents who exceed these recommendations has increased from approximately 10% in 1997 to 36% in 2006 [13]. Unfortunately, despite acknowledgement of the health consequences of childhood obesity [14,15,16] and increasing interest in the possible relationship between screen-based SB and body composition, no study has examined the independent effect of screen time on body mass distribution among Chinese children and adolescents. Moreover, little is known about the association between body mass distribution and non-screen-based SB, e.g., homework, on which 64.5% of Chinese children reportedly spent one or more hours per day [13].

Therefore, we investigated whether the screen-based (television viewing and computer games) or non-screen-based SB (homework) were associated with percentage body fat (%BF), fat mass index (FMI), and fat-free mass index (FFMI) in a population-based sample of Chinese children and adolescents aged 7–15 years, including moderate-to-vigorous physical activities (MVPA) and EI as confounding factors.

## 2. Materials and Methods 

### 2.1. Study Sample

Children and adolescents aged 7–15 years from four schools (two primary schools and two junior high schools chosen from 35 primary schools and 47 junior high schools, using cluster random sampling with prevalence of obesity as well as distributions of age, gender, and annual family income similar to children in the general population of urban and rural areas in South China [1,17]; 39 classes) of Chengdu, South China were recruited in spring 2013. Details on the participant selection procedure and the study protocol have been described elsewhere [18]. In brief, information on the diet, anthropometry, and life style were collected. The study protocol was approved by the Ethics Committee of West China School of Public Health (No. 4 West China Teaching Hospital) of Sichuan University (Approved date: 5 March 2012). All examinations were performed with the written consent of parents or guardians. 

Initially, 2043 children and adolescents were recruited. Of these, 83 children who were multiple births (which might cause differences in growth and development), 65 children who provided implausible information on energy intake (estimated by energy requirements provided by the Chinese reference specific to age and sex) [19], and 309 children with incomplete data (missing anthropometric data or information on potential confounders) were excluded. Therefore, this analysis is based on a final sample of 1586 participants (798 girls and 788 boys). 

### 2.2. Sedentary Behavior

Information about sedentary behaviors (SB) including television viewing, computer use, and doing homework was collected by a detailed questionnaire similar to a previously validated tool for Chinese children [20,21], which asked about the daily frequency and duration of the corresponding behavior. In the face-to-face interview, children aged nine or older were asked separately for weekdays and weekends, “how many hours do you spend on each of SB (television viewing, computer using, and doing homework) per day?” Homework was defined as work assigned by the teacher and completed on paper (including work assigned by the teacher for children to do in the school). Parents were asked instead if the child was younger than nine. Based on the above data, the mean daily time spent on each of the three SB was estimated in hours by using the following formula: average daily time = ((hours on a weekday) × 5 + (hours on a weekend day) × 2)/7. Screen time (the sum of time spent on television viewing and computer use) was categorized into two groups (less than 2 h per day vs. 2 h per day or more) based on the international screen time recommendations [9,10,11,12]. The proportion of participants who spent more than one or two hours on doing homework was calculated. In addition, to observe the synergistic effect, total sedentary time was initially calculated as the sum of time spent on these three SB.

### 2.3. Physical Activity

Physical activities (PA) inside and outside school settings as well as leisure time activities were self-reported in older children; for younger children parents completed a detailed questionnaire similar to a previously validated tool for Chinese children [20,21]. The questionnaire included a checklist of 38 physical activities, i.e., transport (walking and climbing stairs, etc.), playing/sports (track and field, ball games, dancing, etc.) and chores (dusting, sweeping, tidying room, etc.). Quantitative data on yearly/monthly/weekly/daily frequency and duration of the corresponding PA over the past year were obtained. Daily time of PA was constructed by multiplying frequency and duration and dividing by one year/month/week/day. To quantify the intensity of PA, metabolic equivalent tasks (METs), the value for a particular type of PA representing the ratio of work metabolic rate to a standard resting metabolic rate of 1.0 (4.184 kJ)·kg^−1^·h^−1^) [22], were used according to 2011 update of a published compendium by category [23]. By multiplying the body weight in kg by the METs value and duration of activity, energy expenditure that is specific to a person’s weight was estimated [22]. MVPA were characterized as greater than or equal to 3 METs [24]. Energy expend on MVPA expressed in MJ/day was calculated. 

### 2.4. Anthropometric Measurements

Anthropometric measurements were performed by trained medical workers according to the standard procedures [25], with the participants dressed in underwear only, barefoot, and women’s hair uncovered. With an ultrasonic meter (Weight and Height Instrument DHM-30, Dingheng Ltd., Zhengzhou, China), weight and standing height were measured in duplicate to the nearest 0.1 kg and 0.1 cm, respectively. Skin-fold thicknesses were measured in duplicate on the right side of the body to the nearest 0.1 mm using a caliper (Holtain Ltd., Crosswell, UK). The trained investigators who conducted the measurements undergo regular quality controls. 

For this analysis, %BF was calculated using the equations of Slaughter et al. [26]. Moreover, FMI and FFMI were calculated using following equations [27]: FMI (kg/m^2^) = (weight × %BF)/height^2^; FFMI (kg/m^2^) = (weight × (1 − %BF))/height^2^. Working Group on Obesity in China (WGOC) criteria were used to define children overweight and obesity [28]. Using the Chinese reference curves [29], sex- and age-independent BMI SDS was also calculated.

### 2.5. Energy Intake

Dietary data were collected on three random days within a given individual time frame of 10-days (including two weekdays and one weekend) by trained investigators using a validated 24-h dietary recall [18] in a face-to-face interview. Children aged nine or older were asked to recall all foods and beverages consumed and the corresponding timing [30]; for children younger than nine, parents provided the information on food consumption at home [30] while children provided the dietary intake information from school themselves. Information on recipes, types, and brands of all food items reported was obtained from the interview. Standard serving bowls, plates, and glasses were used to improve the accuracy of the portion size estimates. Dietary intake data from 24-h dietary recall was converted into energy and nutrient intake data using the continuously updated in-house nutrient database (based on the software NCCW: version 11.0, 2014, Qingdao University Medical College) reflecting the China Food Composition [31]. In this analysis, total energy intake (EI) for each child was calculated as individual means of three-day 24-h dietary recall in MJ/day.

### 2.6. Parental Characteristics and Additional Information

A printed questionnaire was scheduled for parents, which included basic information on their children (birth date, birth weight and breast-feeding, etc.) and parental anthropometric measurements (height and weight), as well as socio-demographic data (parental education levels, parental profession, household income, etc.).

Additionally, pubertal stage was considered in this study in consideration of the relatively wide age range in this study sample, which may cover different pubertal stages; chronologic age may be confounded by children of the same age differing considerably in their pubertal stage. Tanner stages for either breast (girls) or genital (boys) development were assessed by one trained investigator in each study office. Pubertal staging is performed according to the standardized criteria published by Tanner [32]. In addition, children and/or parents were asked whether menarche or voice break had already occurred.

### 2.7. Statistical Analysis

SAS procedures (SAS, version 9.3, 2011, SAS Institute Inc., Cary, NC, USA) were used for data analyses. All analyses were performed with significance level at *p* < 0.05, except for interaction tests, where *p* < 0.1 was considered significant. Normality of all continuous variables was examined by using normal probability plots and the Kolmogorov-Smirnov test. Given their non-normality, all continuous variables were presented as median (25th percentile, 75th percentile). Due to differential development of body composition the course of growth in boys and girls and initial analysis indicated a sex differentiated association between SBs and all parameters of body mass distribution (*p* < 0.02), the subsequent analyses in this study was performed for girls and boys separately. 

Spearman’s correlations (PROC CORR in SAS) were used firstly to assess the relations between PA and SB, EI and SB, and PA and EI in the present study. Since the correlations between MVPA energy expenditure (the variable for PA in present analysis) and SB, EI and SB, and MVPA energy expenditure and EI were small (all correlation coefficients <0.08), MVPA energy expenditure and EI were considered concurrently as potential confounding factors.

Sex differences in anthropometric data, SB, EI, and socio-demographic data were tested using the Wilcoxon rank-sum for non-normally distributed continuous variables and the Chi-square test for categorical variables, respectively.

Time spent on screen-based SB was grouped into tertiles (T1–T3) for illustration of its association with general characteristics. Significant differences for non-normally distributed continuous variables were analyzed by Kruskal–Wallis tests, and Chi-square tests were used for categorical variables.

Multivariable linear generalized regression models (PROC GLM in SAS) were used to investigate the associations of SB with %BF, FMI, and FFMI. Screen-based SB, time of doing homework, and total time of these behaviors were defined as the independent variable in separate models. Body mass distribution indexes including %BF, FMI, and FFMI were dependent variables in separate models. The independent and dependent variables that enter the linear regression models were non-normally distributed continuous variables. To improve the fitting effect of the models, log-transformed values of %BF, FMI, and FFMI were used in the models. 

In the basic models, the correlation analyses between each of the SB and Body mass distribution indexes (%BF, FMI, FFMI) were carried out first. In a further step, potential covariates that may affect these associations were added. These included: continuous variables—age (years), birth weight (kg), EI (MJ/day), and MVPA energy expenditure (MJ/day); and categorical variables—pubertal stage [32], breast development in girls (B1–B5) and testicles in boys (<4 mL, ≥4 and <12 mL, ≥12 mL), average annual income of family (<15,000 Yuan, 15,000–35,000 Yuan and >35,000 Yuan) [17], parental overweight (BMI ≥ 24 kg/m^2^) [33], parental education level (<6 years, 6–12 years, and >12 years of schooling), and parental occupation (liberal profession, casual laborer, manual worker, and non-manual worker). Each variable was initially considered separately: only variables that had their own independent significant effect in the basic models or that substantially modified the association of the principal time spent on SB with body mass distribution indexes were included in the subsequent multivariable analyses. The adjusted means were the least-squares means predicted by the model when the other variables were held at their mean values. Then the least-squares means and 95% confidence interval computed by the linear models were back transformed and then presented in the results.

Additionally, to further understand the impact of television viewing, computer use and MVPA energy expenditure on the body mass distribution separately, multivariable linear generalized regression models were also used. 

## 3. Results

General characteristics of the sample in this study stratified by sex are presented in Table 1. Nearly half of our participants were girls (50.3%). Age did not differ between girls and boys, and the median age was 10.0 years; 865 participants (54.5% girls) were aged 7–10 years and 721 (45.5% girls) were between 11 and 15 years old. Boys spent slightly more energy on MVPA, but also more time on screen-based SB and homework than girls. Of the study sample, 43.0% boys and 33.0% girls spent more than 2 h per day on screen time. 

Family and anthropometric characteristics of the girls and boys are presented by tertiles of screen time in Table 2. Girls with the highest screen time had a significantly higher percentage of maternal overweight and higher %BF, FMI, and FFMI compared to girls in the lowest tertile of screen time. Among boys, paternal educational level, total energy intake, FMI, and FFMI were significantly different by tertiles. 

**Figure 1:**
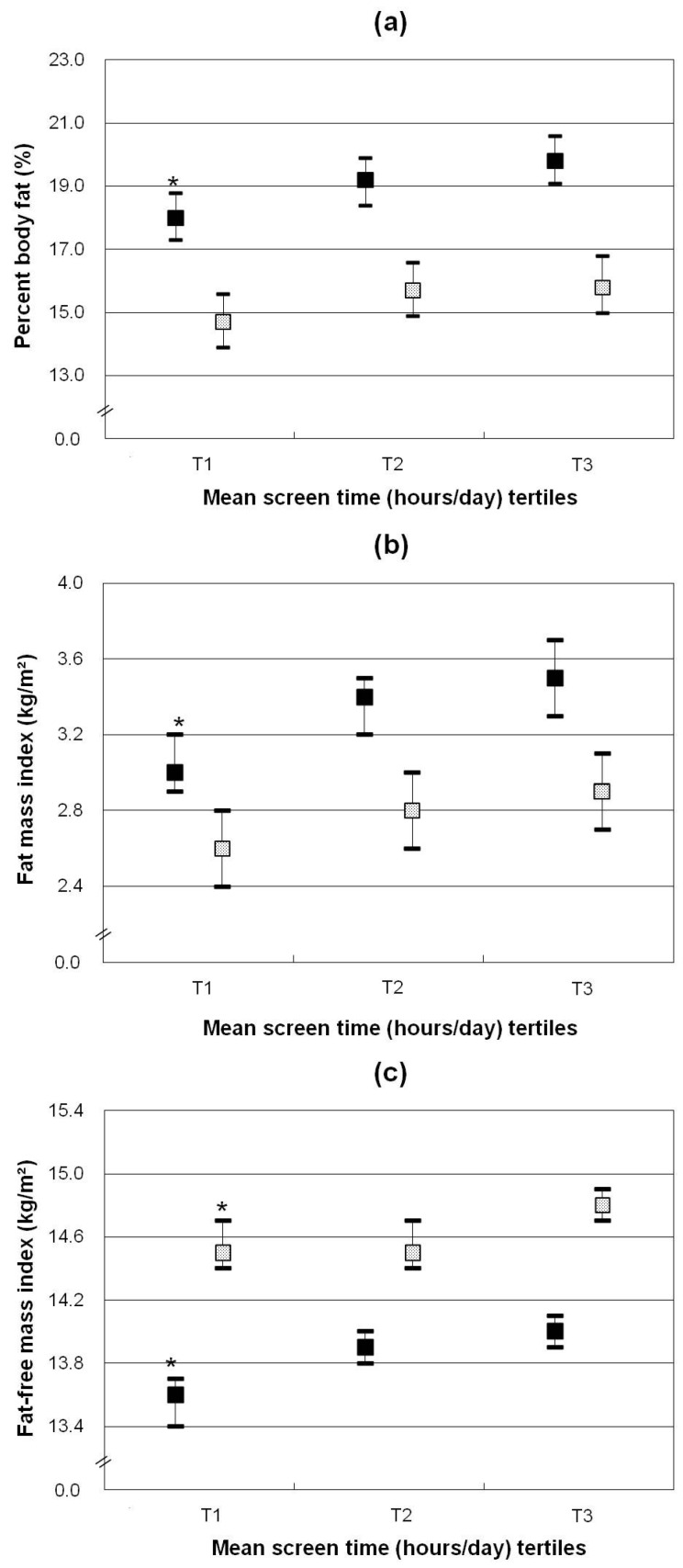
Body mass indicators ((**a**) percentage body fat; (**b**) fat mass index; (**c**) fat-free mass index) by tertiles of time spent on screen-based sedentary behaviors (h/day) of children stratified by gender (girls ■, boys 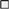). Data shown are least-squares means (95% CI) adjusted for average annual income of family, maternal overweight, total energy intake (MJ/day) and MVPA energy expenditure (MJ/day)). * *p* for trend <0.05. *p* for trend refers to *p* values obtained by linear regression models with total time spent on sedentary behaviors as continuous variables.

After adjusting for maternal overweight, the average annual income of family, MVPA energy expenditure and/or EI, screen-based sedentary time was positively related to all parameters of body mass distribution among girls, i.e., %BF, FMI and FFMI (all *p* values < 0.03) (Table 3 and Figure 1). Girls in the highest screen time tertile had 16.7% relative higher FMI and 2.9% relative higher FFMI than girls in the lowest tertile. Among boys, screen time was positively related to FMI and FFMI, after adjustment for maternal overweight, the average annual income of family, and MVPA energy expenditure. However, after further adjusting for EI, the association between screen time and FMI was no longer statistically significant (*p* > 0.09), while the association with FFMI remained significant (*p* < 0.03). 

**Figure 2:**
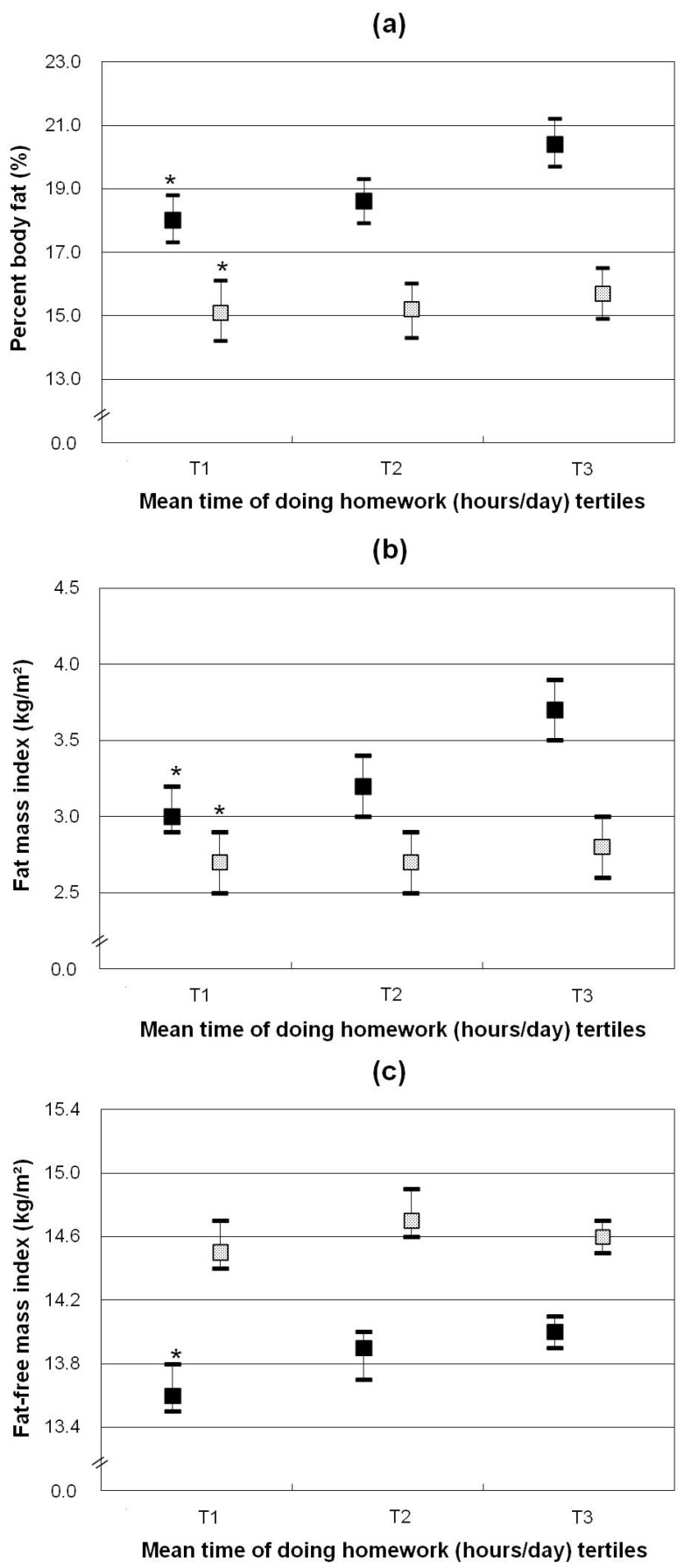
Body mass indicators ((**a**) percentage body fat; (**b**) fat mass index; (**c**) fat-free mass index) by tertiles of total time spent on doing homework (h/day) of children stratified by gender (girls ■, boys 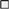). Data shown are least-squares means (95% CI) adjusted for average annual income of family, maternal overweight, total energy intake (MJ/day) and MVPA energy expenditure (MJ/day)). * *p* for trend <0.05. *p* for trend refers to *p* values obtained by linear regression models with total time spent on sedentary behaviors as continuous variables.

Table 4 and Figure 2 show the association between time spent doing homework and body mass distribution measures. Time spent doing homework was positively and independently related to %BF, FMI, and FFMI in girls (all *p* values < 0.03). Girls who spent more time doing homework (in the highest tertile) had 23.3% relative higher FMI and 2.9% relative higher FFMI than those with less time for homework (in the lowest tertile). In boys, time doing homework was positively associated with %BF and FMI (*p* = 0.03), but not associated with FFMI.

**Figure 3:**
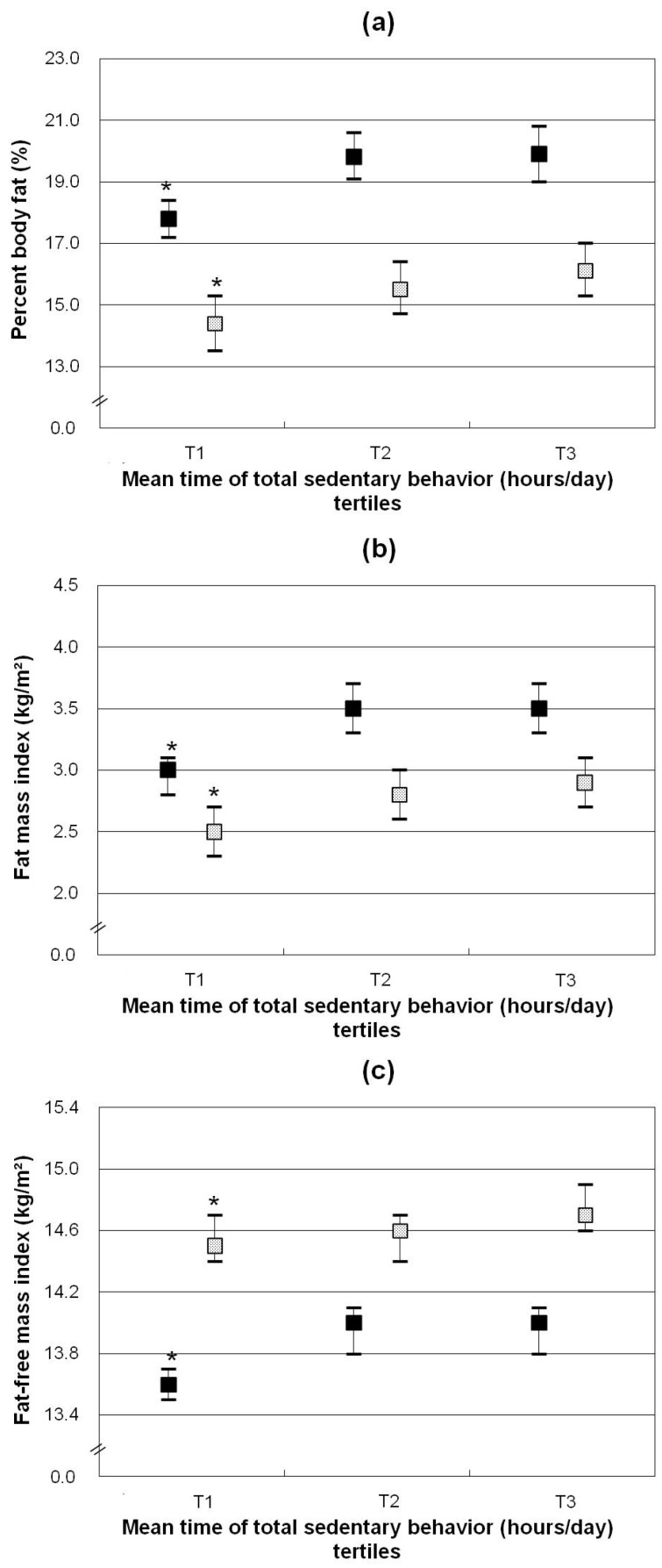
Body mass indicators ((**a**) percentage body fat; (**b**) fat mass index; (**c**) fat-free mass index) by tertiles of total time spent on sedentary behaviors (h/day) of children stratified by gender (girls ■, boys 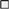). Data shown are least-squares means (95% CI) adjusted for average annual income of family, maternal overweight, total energy intake (MJ/day) and MVPA energy expenditure (MJ/day)). * *p* for trend <0.05. *p* for trend refers to *p* values obtained by linear regression models with total time spent on sedentary behaviors as continuous variables.

Associations between time spent on total SB and body mass distribution among girls and boys are shown in Table 5 and Figure 3. In both genders, %BF, FMI, and FFMI were highest in children in the highest SB tertile (all *p* < 0.05). 

**Figure A1:**
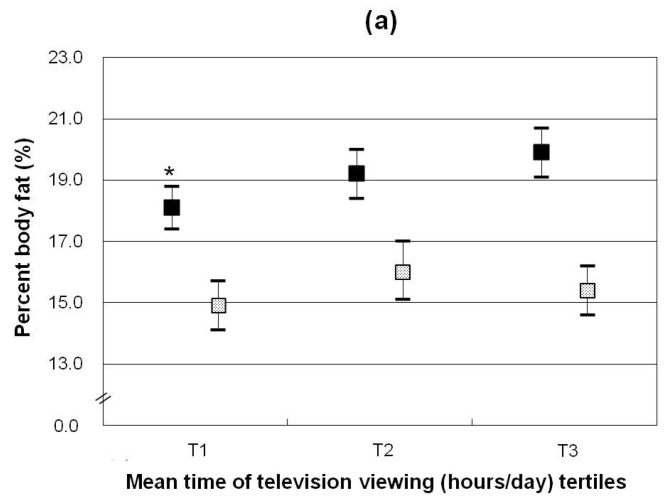

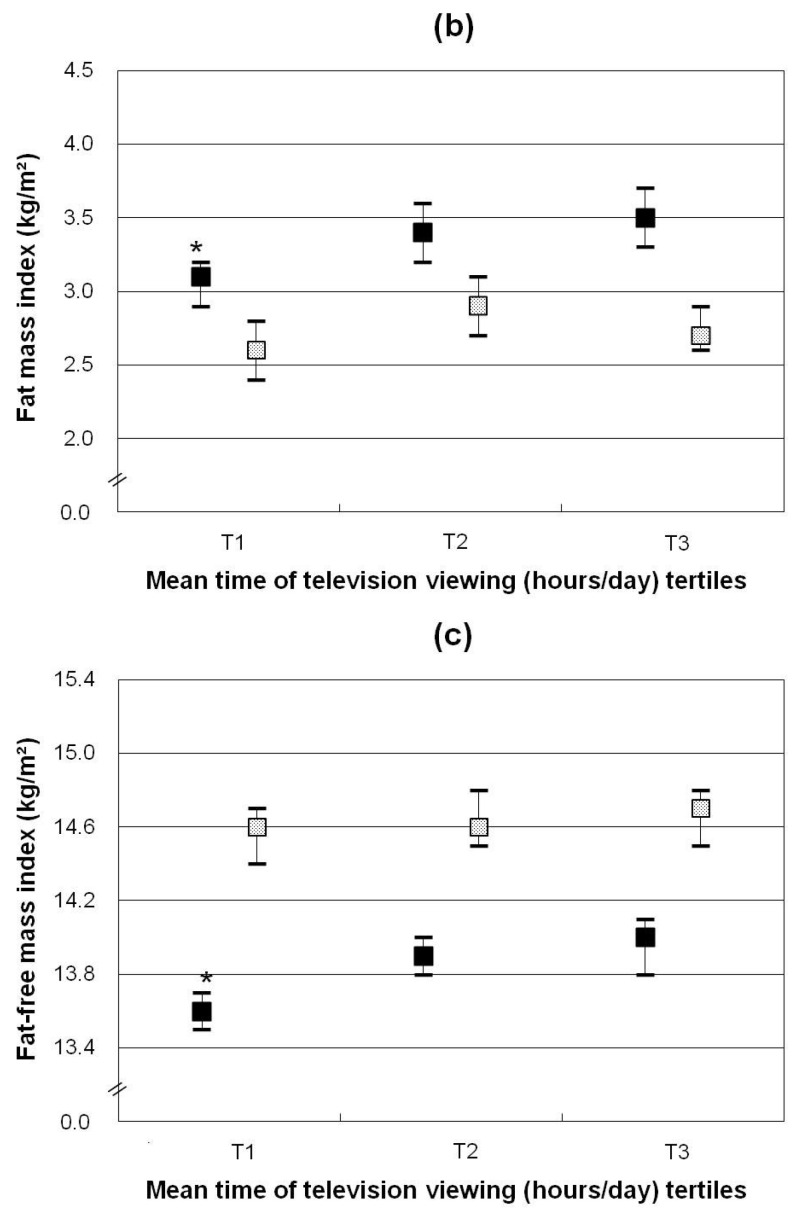
Body mass indicators ((**a**) percentage body fat; (**b**) fat mass index; (**c**) fat-free mass index) by tertiles of time spent on television viewing (h/day) of children stratified by gender (girls ■, boys 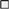). Data shown are least-squares means (95% CI) adjusted for average annual income of family, maternal overweight, total energy intake (MJ/day) and MVPA energy expenditure (MJ/day)); * *p* for trend < 0.05. *p* for trend refers to *p* values obtained by linear regression models with total time spent on sedentary behaviors as continuous variables.

**Figure A2:**
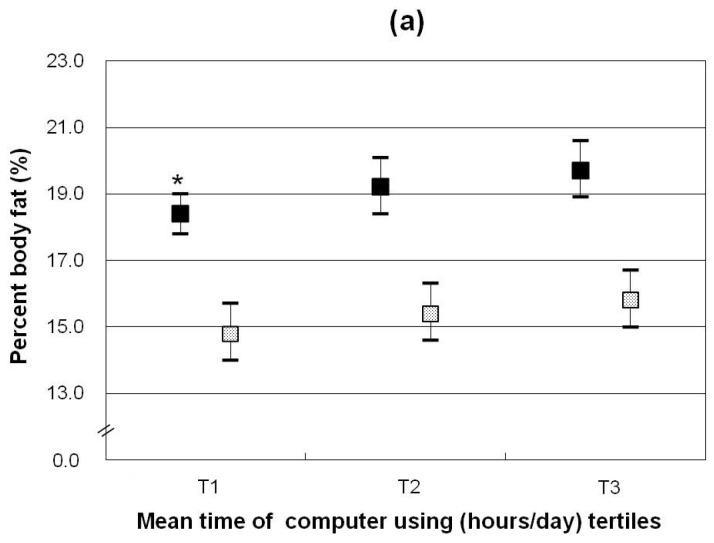

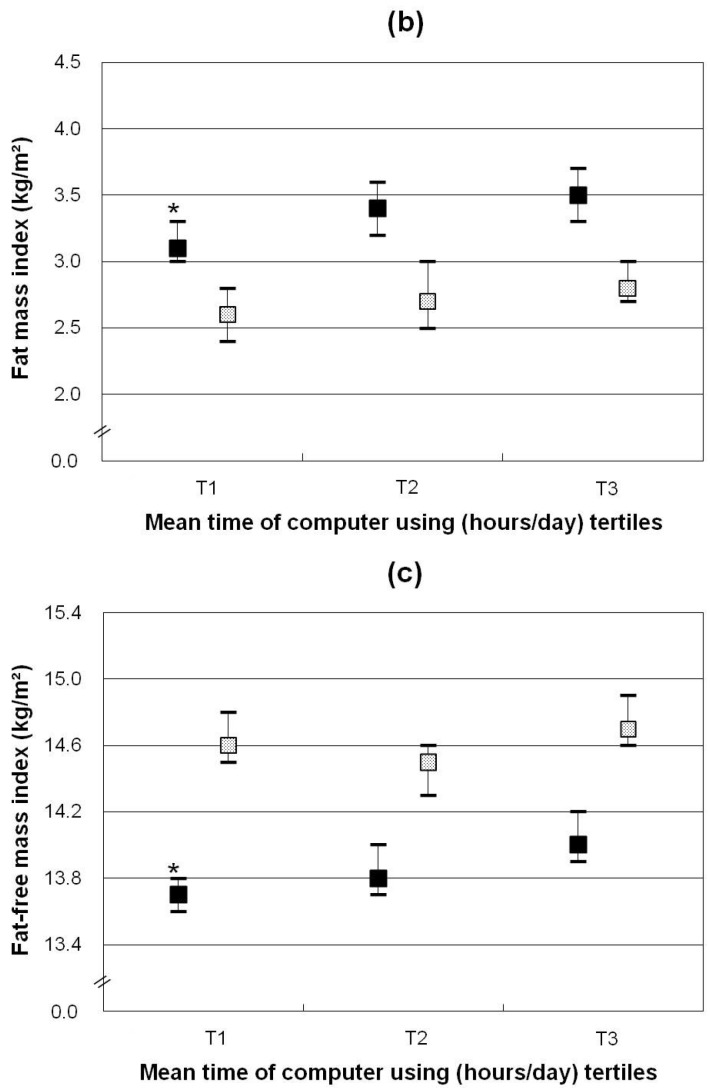
Body mass indicators ((**a**) percentage body fat; (**b**) fat mass index; (**c**) fat-free mass index) by tertiles of time spent on doing homework (h/day) of children stratified by gender (girls ■, boys 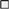); Data shown are least-squares means (95% CI) adjusted for average annual income of family, maternal overweight, total energy intake (MJ/day) and MVPA energy expenditure (MJ/day)); * *p* for trend < 0.05. *p* for trend refers to *p* values obtained by linear regression models with total time spent on sedentary behaviors as continuous variables.

In addition, the relations of television viewing, computer use, and MVPA energy expenditure with body mass distribution were assessed. Time of television viewing and computer use was positively and independently associated with body mass distribution among girls (Appendix Figure A1 and Figure A2). No association was observed between MVPA energy expenditure and body mass distribution in both genders (Appendix Table A1).

## 4. Discussion

The present study suggested that time spent on SB, i.e., screen time and doing homework, was positively associated with body mass distribution in Chinese children and adolescents, independent of PA and EI. Furthermore, these associations were more pronounced in girls. 

The median screen time (television and computer use) observed in the present study (1.6 h/day) was nearly equal to the Chinese national average level of urban children in 2006 (1.6–1.7 h/day) [13], but lower than Western youth in 2009–2013 (2.5–3.5 h/day) [34,35]. The prevalence of spending two or more hours per day on screen time in this study (38.0%) was higher than in previous studies on Chinese children (approximately 36% in 2006) [13], but lower than in Western children (approximately 70% of Australian children in 2005 [36] and 41% of U.S. adolescents in 2013 [35]). In line with the findings of a previous study among Chinese children [13], boys in the current study had more screen time than girls and were less likely to meet the international screen time recommendations. Therefore, our data might indicate that the proportion of children spending much time watching television or using a computer is increasing in China. However, the Western life style has not (yet) fully been adopted as screen time is still more important for Western children. 

In this analysis, 49.5% Chinese children spent one or more hours per day on homework, which was lower than that of the Chinese national survey data (64.5% in 2006) [13], but much higher than that reported by children from Canada (22.9% in 2005–2006) [37]. The differences of time spent on doing homework between Chinese and Western children may lie in an academic-oriented culture [37] and lower economic level in China, especially in South China [17]. 

Albeit many publications focused on the impact of SB on body fat mass among Western children [3,4,5,6,7,8], it remains controversial whether the effects were independent of PA. In addition, none of these studies investigated whether the association of SB with body fat mass was independent of EI. In this analysis, adjustment for EI or PA attenuated the association with FMI at least in boys, which confirms that EI and PA are important confounding factors. Among our participants, screen time was related to body mass indicators, independent of PA and EI. SB has been suggested to be more related to overweight/obese children [38] and overweight/obese children commonly have more fat mass but also more fat-free mass; our findings may thus be partly explained by the proportion of overweight/obese children in the study sample. The complex interplay of SB and life style on body composition among different subgroups cannot be fully capture by statistical methods, hence we cannot preclude a potential contribution of overweight/obese children for which we may have lacked statistical power in this subgroup analysis. 

To date, most studies of Western children [3,4,5,6,7,8] focused on the relationship between SB and fat mass rather than fat-free mass. However, it is unknown whether SB are related to both fat mass and fat-free mass. Despite the potential beneficial impact on fat-free mass, our results showed that SB may be more relevant to fat mass rather than to fat-free mass. Interestingly, time spent on SB among girls was independently and positively related to all body mass distribution indicators in the present study, but not to all indicators among boys. These gender differences may lie in other factors not accounted for in the current analyses (e.g., psychosocial factors) [39,40].

It is well known that childhood obesity is considered one of the most important threats to public health [1], resulting in increasing risk of metabolic disease [14,15,16]. Due to the independent association with body mass distribution and considering the high relevance for fat mass, the time spent on SB among children may be an important modifiable risk factor and addressing this problem may decrease the incidence of obesity and other diseases. In addition, our findings suggested that compared with screen time, the impact of time spent doing homework on body fat mass might be the same or even greater for Chinese children, which indicates that the influence of time spent on SB among children might be culture-specific.

The present study had several considerable strengths, including the large sample size, high participation rate, repetitive anthropometric measurements, and detailed information on SB, PA, and dietary data collected by trained workers in face-to-face interviews. In the present analysis, unlike in other studies, we examined the independence of the association between SB and body mass distribution from PA and EI. A further strength lies in the adjustment for a number of potential confounders related to parental and early life characteristics and socioeconomic status, such as average annual income of family, maternal overweight, and EI. 

Some limitations of the present study should be mentioned. Our study comprised children and adolescents from South China. The non-representativeness of this sample for Chinese children in general may have decreased the generalizability of the findings. However, homogeneity among participants in South China strengthens the internal validity of our findings by maximizing the quality of self-reported activity and reducing residual confounding. Secondly, it was not possible to observe the causality of the effect of SB on body mass distribution because of the cross-sectional design of this study. Thirdly, the %BF was estimated from skin-fold-thickness measurements, which are known to be more susceptible to measurement error than specialized research-based techniques and may underestimate body fat mass [41]. However, intra- and inter-observer variability could be notably reduced when measurements were conducted twice by trained personnel, as was the case in the present study. Furthermore, PA and SB in our study were self-reported and time spent doing homework may not be a good proxy for overall non-screen time. However, these data have been well validated, with high correlations with the measured data [20,21]. In addition, although we adjusted for several covariates, there may be residual confounding due to other variables, such as psychosocial factors, which may explain our findings, at least in part.

## 5. Conclusions 

Our data indicate that sedentary behaviors are positively related to fat mass among Chinese children, independent of physical activities or energy intake. These associations are more pronounced in girls. Further studies should be conducted in overweight/obese participants to confirm our results, and to explore dose-response relationships and causality. Nevertheless, our findings have important implications for public health as they suggest that sedentary behavior may play a significant role in the development and prevention of childhood obesity. Consideration should be given to including strategies to reduce the amount of sedentary time in obesity prevention programs.

## Figures and Tables

**Table 1:** Characteristics ^1^ of the participants by gender in this study (*n* = 1586).

	Girls	Boys	*p*
*n* (%)	798 (50.3)	788 (49.8)	
Age (years)	10.1 (8.6, 11.7)	10.0 (8.6, 11.7)	0.2
Pubertal stage ^2^ (*n*, %)	410 (62.1)	346 (54.8)	0.08
Birth weight (kg)	3.2 (2.9, 3.5)	3.3 (3.0, 3.6)	<0.0001
Family data			
High income of family ^3^ (*n*, %)	193 (24.2)	193 (24.5)	0.9
Maternal overweight ^4^ (*n*, %)	141 (18.4)	143 (19.7)	0.5
Paternal overweight ^4^ (*n*, %)	308 (40.2)	288 (39.8)	0.9
High maternal education level ^5^ (*n*, %)	139 (19.8)	126 (18.1)	0.4
High paternal education level ^5^ (*n*, %)	193 (25.0)	179 (23.3)	0.4
Total energy intake (MJ/day)	7.7 (6.4, 9.5)	8.7 (7.3, 10.7)	<0.0001
MVPA energy expenditure ^6^ (MJ/day)	0.5 (0.3, 0.8)	0.6 (0.3, 0.9)	0.03
Anthropometric data			
BMI SDS ^7^	0.4 (−0.2, 1.0)	0.08 (−0.5, 0.8)	<0.0001
Overweight ^8^ (*n*, %)	104 (13.0)	143 (18.1)	0.01
Percent body fat ^9^ (%)	18.5 (14.9, 24.1)	14.4 (11.1, 20.9)	<0.0001
Fat mass index (kg/m^2^)	3.2 (2.4, 4.5)	2.5 (1.8, 3.9)	<0.0001
Fat-free mass index (kg/m^2^)	13.8 (13.1, 14.6)	14.6 (13.9, 15.3)	<0.0001
Sedentary behaviors			
Screen time ^10^ (h/day)	1.4 (0.8, 2.3)	1.7 (1.0, 2.9)	<0.0001
Screen time ≥2 h/day ^11^ (*n*, %)	263 (33.0)	339 (43.0)	<0.0001
Doing homework (h/day)	0.8 (0.5, 1.5)	1.1 (0.8, 1.8)	<0.0001
Doing homework ≥1 h/day (*n*, %)	359 (45.0)	426 (54.0)	0.0004
Doing homework ≥2 h/day (*n*, %)	114 (14.3)	136 (17.2)	0.1

^1^ Values are median (25th percentile, 75th percentile) or frequencies. Test for difference between boys and girls was performed by using Wilcoxon rank-sum for non-normally distributed continuous variables and chi-square test for categorical variables; ^2^ Pubertal stages (puberty onset: Tanner stage ≥2 for breast development in girls and testicles ≥4 mL in boys) were defined according to the standardized criteria published by Tanner [32]; ^3^ Average annual income of family at least ≥35,000 CNY (Chinese Yuan), which is moderate level among the general population in South China [17]; ^4^ BMI (in kg/m^2^) ≥24 [33]; ^5^ At least 12 years of school education; ^6^ MVPA energy expenditure, energy expended on moderate-to-vigorous physical activities (MJ/day) [23]; ^7^ BMI SDS, body mass index standard deviation score was calculated according to Chinese children reference curve [29]; ^8^ Calculated according to the WGOC criteria [28]; ^9^ Calculated according to Slaughter equations [26]; ^10^ Screen time were the sum of time spent on television viewing and computer use; ^11^ Screen time were categorized into two groups (<2 h/day and ≥2 h/day) based on the international screen time recommendations [9,10,11,12].

**Table 2:** Characteristics ^1^ of study sample by screen time tertiles ^2^ (h/day).

	Daily Time Spent on Screen Time			*p*
	T1	T2	T3	
Girls				
*n*	251	284	263	
Age (years)	9.4 (8.1, 10.9)	9.9 (8.5, 11.7)	11.0 (9.5, 12.3)	0.03
Pubertal stage ^3^ (*n*, %)	97 (49.7)	147 (64.2)	166 (70.3)	0.06
Birth weight (kg)	3.2 (2.9, 3.5)	3.2 (2.9, 3.5)	3.3 (3.0, 3.5)	0.4
Family data				
High income of family ^4^ (*n*, %)	62 (24.7)	59 (20.8)	72 (27.4)	0.2
Maternal overweight ^5^ (*n*, %)	30 (12.3)	55 (20.2)	56 (22.2)	0.01
Paternal overweight ^5^ (*n*, %)	95 (39.3)	99 (36.8)	114 (44.7)	0.2
High maternal education level ^6^ (*n*, %)	52 (23.2)	44 (17.7)	43 (18.6)	0.3
High paternal education level ^6^ (*n*, %)	72 (29.6)	63 (23.0)	58 (22.8)	0.1
Total energy intake (MJ/day)	7.4 (6.3, 9.7)	7.6 (6.3, 9.4)	8.0 (6.6, 9.6)	0.2
MVPA energy expenditure ^7^ (MJ/day)	0.4 (0.3, 0.7)	0.4 (0.2, 0.7)	0.5 (0.3, 0.9)	0.1
Anthropometric data				
BMI SDS ^8^	0.2 (−0.3, 0.9)	0.5 (−0.2, 1.1)	0.4 (−0.1, 1.0)	0.07
Overweight ^9^ (*n*, %)	25 (10.0)	45 (15.8)	34 (12.9)	0.09
Percent body fat ^10^ (%)	17.2 (13.9, 23.7)	18.7 (18.9, 24.0)	19.7 (16.0, 24.5)	0.0007
Fat mass index (kg/m^2^)	2.8 (2.1, 4.3)	3.2 (2.4, 4.4)	3.5 (2.6, 4.7)	<0.0001
Fat-free mass index (kg/m^2^)	13.6 (12.9, 14.3)	13.8 (13.1, 14.7)	14.1 (13.3, 14.8)	<0.0001
Boys				
*n*	263	262	263	
Age (years)	9.6 (8.3, 11.1)	10.5 (8.7, 12.0)	11.0 (9.4, 12.5)	0.04
Pubertal stage ^3^ (*n*, %)	110 (53.4)	125 (57.6)	111 (53.4)	0.6
Birth weight (kg)	3.3 (3.0, 3.8)	3.4 (3.0, 3.6)	3.3 (3.0, 3.5)	0.7
Family data				
High income of family ^4^ (*n*, %)	56 (21.3)	62 (23.7)	75 (28.5)	0.1
Maternal overweight ^5^ (*n*, %)	46 (33.3)	40 (33.9)	57 (32.8)	0.1
Paternal overweight ^5^ (*n*, %)	99 (41.6)	96 (39.3)	93 (38.4)	0.8
High maternal education level ^6^ (*n*, %)	49 (20.9)	44 (19.1)	33 (14.2)	0.2
High paternal education level ^6^ (*n*, %)	61 (23.7)	74 (29.2)	44 (17.2)	0.007
Total energy intake (MJ/day)	8.4 (7.1, 10.3)	8.6 (7.0, 10.6)	9.1 (7.6, 11.2)	0.0002
MVPA energy expenditure ^7^ (MJ/day)	0.5 (0.3, 0.9)	0.4 (0.2, 0.8)	0.5 (0.3, 0.8)	0.2
Anthropometric data				
BMI SDS ^8^	0.01 (−0.5, 0.7)	0.1 (−0.5, 0.8)	0.07 (−0.5, 0.8)	1.0
Overweight ^9^ (*n*, %)	46 (17.5)	47 (17.9)	50 (19.0)	0.7
Percent body fat ^10^ (%)	13.9 (10.7, 19.6)	14.5 (11.1, 21.2)	15.1 (11.8, 21.7)	0.055
Fat mass index (kg/m^2^)	2.4 (1.7, 3.7)	2.6 (1.8, 4.0)	2.7 (1.9, 4.0)	0.03
Fat-free mass index (kg/m^2^)	14.5 (13.8, 15.2)	14.5 (13.9, 15.2)	14.8 (14.2, 15.5)	0.0006

^1^ Values are median (25th percentile, 75th percentile) or frequencies. Test for difference between tertiles of screen time was performed by using Kruskal–Wallis tests for non-normally distributed continuous variables chi-square test for categorical variables; ^2^ Screen time (in h/day) were the sum of time spent on television viewing and computer use; ^3^ Pubertal stages (puberty onset: Tanner stage ≥2 for breast development in girls and testicles ≥4 mL in boys) were defined according to the standardized criteria published by Tanner [32]; ^4^ Average annual income of family at least ≥35,000 CNY (Chinese Yuan), which is moderate level among the general population in South China [17]; ^5^ BMI (in kg/m^2^) ≥24 [33]; ^6^ At least 12 years of school education; ^7^ MVPA energy expenditure, energy expended on moderate-to-vigorous physical activities (MJ/day) [23]; ^8^ BMI SDS, body mass index standard deviation score was calculated according to Chinese children reference curve [29]; ^9^ Calculated according to the WGOC criteria [28]; ^10^ Calculated according to Slaughter equations [26].

**Table 3:** Multiple linear regression least-squares means and 95% confidence interval for the association of screen time (h/day) with percentage body fat, fat mass index, and fat-free mass index (*n* = 1586) ^1^.

	Tertiles of Daily Time Spent on Screen ^2^			*p* for Trend
	T1	T2	T3	
Girls (*n* = 797)	0.6 (0.4, 0.8) ^3^	1.4 (1.1, 1.6) ^3^	2.9 (2.3, 3.9) ^3^	
Percentage body fat (%)				
Unadjusted model	18.0 (17.3, 18.7)	19.1 (18.4, 19.9)	20.1 (19.4, 20.9)	0.0004
Model A ^4^	18.0 (17.3, 18.7)	19.1 (18.3, 19.8)	20.0 (19.2, 20.9)	0.0006
Model B ^5^	18.0 (17.3, 18.8)	19.2 (18.4, 20.0)	19.8 (19.1, 20.6)	0.03
Model C ^6^	18.0 (17.3, 18.7)	19.1 (18.3, 19.8)	20.0 (19.2, 20.8)	0.0007
Model D ^7^	18.0 (17.3, 18.8)	19.2 (18.4, 19.9)	19.8 (19.1, 20.6)	0.003
Fat mass index (kg/m^2^)				
Unadjusted model	3.0 (2.9, 3.2)	3.3 (3.2, 3.5)	3.6 (3.4, 3.8)	0.0001
Model A ^4^	3.0 (2.9, 3.2)	3.3 (3.1, 3.5)	3.6 (3.4, 3.8)	0.0002
Model B ^5^	3.0 (2.9, 3.2)	3.4 (3.2, 3.5)	3.5 (3.3, 3.7)	0.001
Model C ^6^	3.0 (2.9, 3.2)	3.3 (3.1, 3.5)	3.6 (3.4, 3.8)	0.0002
Model D ^7^	3.0 (2.9, 3.2)	3.4 (3.2, 3.5)	3.5 (3.3, 3.7)	0.001
Fat-free mass index (kg/m^2^)				
Unadjusted model	13.6 (13.4, 13.7)	13.9 (13.8, 14.0)	14.1 (13.9, 14.2)	<0.0001
Model A ^4^	13.6 (13.4, 13.7)	13.9 (13.7, 14.0)	14.0 (13.9, 14.2)	<0.0001
Model B ^5^	13.6 (13.4, 13.7)	13.9 (13.8, 14.0)	14.0 (13.9, 14.1)	0.0001
Model C ^6^	13.6 (13.4, 13.7)	13.9 (13.7, 14.0)	14.0 (13.9, 14.2)	<0.0001
Model D ^7^	13.6 (13.4, 13.7)	13.9 (13.8, 14.0)	14.0 (13.9, 14.1)	0.0001
Boys (*n* = 789)	0.8 (0.5, 0.8) ^3^	1.4 (1.1, 1.6) ^3^	3.6 (2.9, 4.6) ^3^	
Percentage body fat (%)				
Unadjusted model	14.5 (13.8, 15.4)	15.6 (14.8, 16.5)	15.9 (15.1, 16.8)	0.04
Model A ^4^	14.6 (13.8, 15.4)	15.7 (14.8, 16.6)	15.9 (15.1, 16.9)	0.058
Model B ^5^	14.6 (13.8, 15.4)	15.7 (14.8, 16.6)	15.9 (15.1, 16.9)	0.058
Model C ^6^	14.7 (13.9, 15.6)	15.7 (14.9, 16.6)	15.8 (15.0, 16.8)	0.1
Model D ^7^	14.7 (13.9, 15.6)	15.7 (14.9, 16.6)	15.8 (15.0, 16.8)	0.1
Fat mass index (kg/m^2^)				
Unadjusted model	2.5 (2.4, 2.7)	2.8 (2.6, 3.0)	2.9 (2.7, 3.1)	0.03
Model A ^4^	2.5 (2.4, 2.7)	2.8 (2.6, 3.0)	2.9 (2.7, 3.1)	0.04
Model B ^5^	2.5 (2.4, 2.7)	2.8 (2.6, 3.0)	2.9 (2.7, 3.1)	0.04
Model C ^6^	2.6 (2.4, 2.8)	2.8 (2.6, 3.0)	2.9 (2.7, 3.1)	0.09
Model D ^7^	2.6 (2.4, 2.8)	2.8 (2.6, 3.0)	2.9 (2.7, 3.1)	0.09
Fat-free mass index (kg/m^2^)				
Unadjusted model	14.5 (14.4, 14.7)	14.6 (14.5, 14.7)	14.8 (14.7, 15.0)	0.001
Model A ^4^	14.5 (14.4, 14.6)	14.5 (14.4, 14.7)	14.8 (14.7, 14.9)	0.001
Model B ^5^	14.5 (14.4, 14.6)	14.5 (14.4, 14.7)	14.8 (14.7, 14.9)	0.001
Model C ^6^	14.5 (14.4, 14.6)	14.5 (14.4, 14.7)	14.8 (14.7, 14.9)	0.003
Model D ^7^	14.5 (14.4, 14.7)	14.5 (14.4, 14.7)	14.8 (14.7, 14.9)	0.003

^1^ Values are models adjusted least-squares means and 95% confidence interval. Linear trends (*p* for trend) were obtained with time spent on screen time as continuous variables; ^2^ Ranges for tertiles (T) 1 through 3; ^3^ Values are median (25th percentile, 75th percentile) of screen time (in h/day); ^4^ Model A: adjusted for average annual income of family and maternal overweight; ^5^ Model B: as model A and additionally adjusted for MVPA energy expenditure (MJ/day); ^6^ Model C: as model A and additionally adjusted for total energy intake (MJ/day); ^7^ Model D: as model A and additionally adjusted for total energy intake (MJ/day) and MVPA energy expenditure (MJ/day).

**Table 4:** Multiple linear regression least-squares means and 95% confidence interval for the association of doing homework (h/day) with percentage body fat, fat mass index, and fat-free mass index (*n* = 1586) ^1^.

	Tertiles of Daily Time Spent on Doing Homework ^2^			*p* for Trend
	T1	T2	T3	
Girls (*n* = 797)	0.5 (0.5, 0.5) ^3^	0.8 (0.8, 1.1) ^3^	1.8 (1.5, 2.5) ^3^	
Percentage body fat (%)				
Unadjusted model	17.8 (17.1, 18.5)	18.8 (18.1, 19.6)	20.7 (19.9, 21.5)	0.0007
Model A ^4^	17.7 (17.0, 18.5)	18.7 (18.0, 19.5)	20.6 (19.8, 21.4)	0.001
Model B ^5^	18.4 (17.8, 19.0)	19.2 (18.4, 20.1)	19.8 (18.9, 20.6)	0.003
Model C ^6^	17.7 (17.0, 18.4)	18.7 (18.0, 19.5)	20.6 (19.8, 21.4)	0.0009
Model D ^7^	18.0 (17.3, 18.7)	18.6 (17.9, 19.3)	20.4 (19.7, 21.2)	0.003
Fat mass index (kg/m^2^)				
Unadjusted model	3.0 (2.8, 3.2)	3.3 (3.1, 3.5)	3.7 (3.5, 3.9)	0.0005
Model A ^4^	3.0 (2.8, 3.1)	3.3 (3.1, 3.5)	3.7 (3.5, 3.9)	0.0007
Model B ^5^	3.1 (3.0, 3.3)	3.4 (3.2, 3.6)	3.5 (3.3, 3.7)	0.002
Model C ^6^	3.0 (2.8, 3.1)	3.3 (3.1, 3.4)	3.7 (3.5, 3.9)	0.0006
Model D ^7^	3.0 (2.9, 3.2)	3.2 (3.0, 3.4)	3.7 (3.5, 3.9)	0.003
Fat-free mass index (kg/m^2^)				
Unadjusted model	13.6 (13.5, 13.8)	13.9 (13.8, 14.1)	14.1 (13.9, 14.2)	0.02
Model A ^4^	13.6 (13.4, 13.7)	13.9, 13.8. 14.0	14.0 (13.9, 14.2)	0.01
Model B ^5^	13.7 (13.6, 13.8)	13.8 (13.7, 14.0)	14.0 (13.9, 14.2)	0.03
Model C ^6^	13.6 (13.4, 13.7)	13.9 (13.7, 14.0)	14.0 (13.9, 14.2)	0.01
Model D ^7^	13.6 (13.5, 13.8)	13.9 (13.7, 14.0)	14.0 (13.9, 14.1)	0.03
Boys (*n* = 789)	0.5 (0.5, 0.5) ^3^	0.8 (0.8, 1.1) ^3^	1.8 (1.5, 2.5) ^3^	
Percentage body fat (%)				
Unadjusted model	14.8 (13.8, 15.7)	15.1 (14.3, 16.0)	15.9 (15.2, 16.7)	0.03
Model A ^4^	14.8 (13.8, 15.8)	15.1 (14.3, 16.0)	16.0 (15.2, 16.8)	0.03
Model B ^5^	14.6 (13.7, 15.5)	15.6 (14.7, 16.5)	15.9 (15.1, 16.8)	0.03
Model C ^6^	14.8 (13.9, 15.8)	15.2 (14.3, 16.1)	15.9 (15.1, 16.7)	0.03
Model D ^7^	15.1 (14.2, 16.1)	15.2 (14.3, 16.0)	15.7 (14.9, 16.5)	0.04
Fat mass index (kg/m^2^)				
Unadjusted model	2.6 (2.4, 2.8)	2.7 (2.5, 2.9)	2.8 (2.7, 3.0)	0.03
Model A ^4^	2.6 (2.4, 2.8)	2.7 (2.5, 2.9)	2.8 (2.7, 3.0)	0.03
Model B ^5^	2.6 (2.4, 2.8)	2.7 (2.5, 3.0)	2.9 (2.7, 3.1)	0.03
Model C ^6^	2.6 (2.4, 2.8)	2.7 (2.5, 2.9)	2.8 (2.7, 3.0)	0.03
Model D ^7^	2.7 (2.5, 2.9)	2.7 (2.5, 2.9)	2.8 (2.6, 3.0)	0.03
Fat-free mass index (kg/m^2^)				
Unadjusted model	14.6 (14.4, 14.7)	14.8 (14.6, 14.9)	14.6 (14.5, 14.7)	0.4
Model A ^4^	14.5 (14.4, 14.7)	14.7 (14.6, 14.9)	14.6 (14.5, 14.7)	0.5
Model B ^5^	14.6 (14.4, 14.7)	14.5 (14.3, 14.6)	14.7 (14.6, 14.9)	0.5
Model C ^6^	14.5 (14.4, 14.7)	14.7 (14.6, 14.9)	14.6 (14.5, 14.7)	0.5
Model D ^7^	14.5 (14.4, 14.7)	14.7 (14.6, 14.9)	14.6 (14.5, 14.7)	0.5

^1^ Values are models adjusted least-squares means and 95% confidence interval. Linear trends (*p* for trend) were obtained with time spent on doing homework as continuous variables; ^2^ Ranges for tertiles (T) 1 through 3; ^3^ Values are median (25th percentile, 75th percentile) of screen time (in h/day); ^4^ Model A: adjusted for average annual income of family and maternal overweight; ^5^ Model B: as model A and additionally adjusted for MVPA energy expenditure (MJ/day); ^6^ Model C: as model A and additionally adjusted for total energy intake (MJ/day); ^7^ Model D: as model A and additionally adjusted for total energy intake (MJ/day) and MVPA energy expenditure (MJ/day).

**Table 5:** Multiple linear regression least-squares means and 95% confidence interval for the association of total sedentary time (h/day) with percentage body fat, fat mass index, and fat-free mass index (*n* = 1586) ^1^.

	Tertiles of Daily Time Spent on Total Sedentary Behavior ^2^			*p* for Trend
	T1	T2	T3	
Girls (*n* = 797)	1.5 (1.2, 1.9) ^3^	2.7 (2.5, 3.1) ^3^	4.7 (4.1, 5.8) ^3^	
Percentage body fat (%)				
Unadjusted model	17.7 (17.1, 18.4)	19.8 (19.0, 20.6)	20.2 (19.4, 21.1)	<0.0001
Model A ^4^	17.7 (17.1, 18.4)	19.7 (19.0, 20.5)	20.1 (19.2, 21.0)	<0.0001
Model B ^5^	17.8 (17.2, 18.4)	19.8 (19.1, 20.6)	19.9 (19.0, 20.8)	0.0002
Model C ^6^	17.7 (17.1, 18.4)	19.7 (19.0, 20.5)	20.1 (19.2, 21.0)	<0.0001
Model D ^7^	17.8 (17.2, 18.4)	19.8 (19.1, 20.6)	19.9 (19.0, 20.8)	0.0002
Fat mass index (kg/m^2^)				
Unadjusted model	3.0 (2.8, 3.1)	3.5 (3.3, 3.7)	3.6 (3.4, 3.9)	<0.0001
Model A ^4^	3.0 (2.8, 3.1)	3.5 (3.3, 3.7)	3.6 (3.4, 3.8)	<0.0001
Model B ^5^	3.0 (2.8, 3.2)	3.5 (3.3, 3.7)	3.5 (3.3, 3.7)	<0.0001
Model C ^6^	3.0 (2.8, 3.1)	3.5 (3.3, 3.7)	3.6 (3.4, 3.8)	<0.0001
Model D ^7^	3.0 (2.8, 3.1)	3.5 (3.3, 3.7)	3.5 (3.3, 3.7)	<0.0001
Fat-free mass index (kg/m^2^)				
Unadjusted model	13.6 (13.5, 13.7)	14.0 (13.9, 14.1)	14.1 (13.9, 14.2)	<0.0001
Model A ^4^	13.6 (13.5, 13.7)	14.0 (13.8, 14.1)	14.0 (13.9, 14.2)	<0.0001
Model B ^5^	13.6 (13.5, 13.7)	14.0 (13.8, 14.1)	14.0 (13.8, 14.1)	<0.0001
Model C ^6^	13.6 (13.5, 13.7)	14.0 (13.8, 14.1)	14.0 (13.9, 14.2)	<0.0001
Model D ^7^	13.6 (13.5, 13.7)	14.0 (13.8, 14.1)	14.0 (13.8, 14.1)	<0.0001
Boys (*n* = 789)	1.6 (1.3, 1.9) ^3^	2.8 (2.5, 3.2) ^3^	5.0 (4.2, 5.9) ^3^	
Percentage body fat (%)				
Unadjusted model	14.2 (13.4, 15.1)	15.5 (14.6, 16.4)	16.2 (15.4, 17.0)	0.008
Model A ^4^	14.2 (13.4, 15.1)	15.5 (14.7, 16.5)	16.2 (15.3, 17.1)	0.01
Model B ^5^	14.2 (13.4, 15.1)	15.6 (14.7, 16.5)	16.2 (15.3, 17.1)	0.01
Model C ^6^	14.4 (13.5, 15.3)	15.5 (14.7, 16.4)	16.1 (15.3, 17.0)	0.02
Model D ^7^	14.4 (13.5, 15.3)	15.5 (14.7, 16.4)	16.1 (15.3, 17.0)	0.02
Fat mass index (kg/m^2^)				
Unadjusted model	2.5 (2.3, 2.7)	2.8 (2.6, 3.0)	2.9 (2.7, 3.1)	0.005
Model A ^4^	2.5 (2.3, 2.7)	2.8 (2.6, 3.0)	2.9 (2.7, 3.1)	0.007
Model B ^5^	2.5 (2.3, 2.7)	2.8 (2.6, 3.0)	2.9 (2.7, 3.1)	0.007
Model C ^6^	2.5 (2.3, 2.7)	2.8 (2.6, 3.0)	2.9 (2.7, 3.1)	0.02
Model D ^7^	2.5 (2.3, 2.7)	2.8 (2.6, 3.0)	2.9 (2.7, 3.1)	0.02
Fat-free mass index (kg/m^2^)				
Unadjusted model	14.5 (14.4, 14.7)	14.6 (14.5, 14.7)	14.8 (14.7, 14.9)	0.001
Model A ^4^	14.5 (14.4, 14.6)	14.6 (14.4, 14.7)	14.7 (14.6, 14.9)	0.002
Model B ^5^	14.5 (14.4, 14.6)	14.6 (14.4, 14.7)	14.7 (14.6, 14.9)	0.002
Model C ^6^	14.5 (14.4, 14.7)	14.6 (14.4, 14.7)	14.7 (14.6, 14.9)	0.005
Model D ^7^	14.5 (14.4, 14.7)	14.6 (14.4, 14.7)	14.7 (14.6, 14.9)	0.005

^1^ Values are models adjusted least-squares means and 95% confidence interval. Linear trends (*p* for trend) were obtained with time spent on total sedentary behavior as continuous variables; ^2^ Ranges for tertiles (T) 1 through 3; ^3^ Values are median (25th percentile, 75th percentile) of screen time (in h/day); ^4^ Model A: adjusted for average annual income of family and maternal overweight; ^5^ Model B: as model A and additionally adjusted for MVPA energy expenditure (MJ/day); ^6^ Model C: as model A and additionally adjusted for total energy intake (MJ/day); ^7^ Model D: as model A and additionally adjusted for total energy intake (MJ/day) and MVPA energy expenditure (MJ/day).

## Appendix A

**Table A1:** Multiple linear regression least-squares means and 95% confidence interval for the association of moderate-to-vigorous physical activity^1^ with percentage body fat, fat mass index and fat-free mass index (*n* = 1586).

	Percentage Body Fat (%)		Fat Mass Index (kg/m^2^)		Fat-Free Mass Index (kg/m^2^)	
	Estimate (SE)	*p*	Estimate (SE)	*p*	Estimate (SE)	*p*
Girls (*n* = 797)						
Unadjusted model	−0.0001 (0.0004)	0.7	−0.00006 (0.0005)	0.9	0.00005 (0.00009)	0.6
Model A ^2^	−0.0002 (0.0004)	0.7	−0.0001 (0.0005)	0.8	0.00006 (0.00009)	0.5
Model B ^3^	−0.0002 (0.0004)	0.6	−0.0001 (0.0005)	0.9	0.00005 (0.00009)	0.5
Model C ^4^	−0.0002 (0.0004)	0.5	−0.0002 (0.0005)	0.7	0.00004 (0.00009)	0.6
Model D ^5^	−0.0002 (0.0004)	0.5	−0.0002 (0.0005)	0.7	0.00004 (0.00009)	0.6
Boys (*n* = 789)						
Unadjusted model	−0.00003 (0.0003)	0.9	−0.00005 (0.0004)	0.9	−0.00003 (0.00005)	0.6
Model A ^2^	−0.00003 (0.0003)	0.9	−0.00005 (0.0004)	0.9	−0.00002 (0.00005)	0.7
Model B ^3^	−0.00005 (0.0003)	0.9	−0.00008 (0.0004)	0.9	−0.00003 (0.00005)	0.6
Model C ^4^	−0.000008 (0.0003)	1.0	−0.00002 (0.0004)	1.0	−0.00002 (0.00005)	0.7
Model D ^5^	−0.00003 (0.0003)	0.9	−0.00004 (0.0004)	0.9	−0.00002 (0.00005)	0.7

^1^ moderate-to-vigorous physical activity energy expenditure (expressed in MJ/day), the variable for moderate-to-vigorous physical activity in present analysis, is continuous variable; ^2^ Model A: adjusted for average annual income of family and maternal overweight; ^3^ Model B: as model A and additionally adjusted for total sedentary time (h/day); ^4^ Model C: as model A and additionally adjusted for total energy intake (MJ/day); ^5^ Model D: as model A and additionally adjusted for total energy intake (MJ/day) and total time of sedentary behaviors (h/day).

## References

[1] Ji C.Y., Chen T.J., Working Group on Obesity in China (2013). Empirical changes in the prevalence of overweight and obesity among Chinese students from 1985 to 2010 and corresponding preventive strategies. Biomed. Environ. Sci. BES.

[2] Dehghan M., Akhtar-Danesh N., Merchant A.T. (2005). Childhood obesity, prevalence and prevention. Nutr. J..

[3] Jackson D.M., Djafarian K., Stewart J., Speakman J.R. (2009). Increased television viewing is associated with elevated body fatness but not with lower total energy expenditure in children. Am. J. Clin. Nutr..

[4] Davison K.K., Marshall S.J., Birch L.L. (2006). Cross-sectional and longitudinal associations between TV viewing and girls’ body mass index, overweight status, and percentage of body fat. J. Pediatr..

[5] Basterfield L., Pearce M.S., Adamson A.J., Frary J.K., Parkinson K.N., Wright C.M., Reilly J.J., Gateshead Millennium Study Core Team (2012). Physical activity, sedentary behavior, and adiposity in English children. Am. J. Prev. Med..

[6] Steele R.M., van Sluijs E.M., Cassidy A., Griffin S.J., Ekelund U. (2009). Targeting sedentary time or moderate- and vigorous-intensity activity: Independent relations with adiposity in a population-based sample of 10-year-old british children. Am. J. Clin. Nutr..

[7] Ekelund U., Brage S., Froberg K., Harro M., Anderssen S.A., Sardinha L.B., Riddoch C., Andersen L.B. (2006). TV viewing and physical activity are independently associated with metabolic risk in children: The european youth heart study. PLoS Med..

[8] Hjorth M.F., Chaput J.P., Ritz C., Dalskov S.M., Andersen R., Astrup A., Tetens I., Michaelsen K.F., Sjodin A. (2013). Fatness predicts decreased physical activity and increased sedentary time, but not vice versa: Support from a longitudinal study in 8- to 11-year-old children. Int. J. Obes. (Lond.).

[9] Council on Communications and Media (2011). Children, adolescents, obesity, and the media. Pediatrics.

[10] Public Health Agency of Canada Canada’s Physical Activity Guide for Children. http://www.phac-aspc.gc.ca/hp-ps/hl-mvs/pag-gap/cy-ej/index-eng.php.

[11] Australian Government (2005). Australia’s Physical Activity Recommendations for 5–12 Years Old (brochure).

[12] Australian Government (2005). Australia’s Physical Activity Recommendations for 12–18 Years Old (brochure).

[13] Cui Z., Hardy L.L., Dibley M.J., Bauman A. (2011). Temporal trends and recent correlates in sedentary behaviours in Chinese children. Int. J. Behav. Nutr. Phys. Act..

[14] Kelsey M.M., Zaepfel A., Bjornstad P., Nadeau K.J. (2014). Age-related consequences of childhood obesity. Gerontology.

[15] Edwardson C.L., Gorely T., Davies M.J., Gray L.J., Khunti K., Wilmot E.G., Yates T., Biddle S.J. (2012). Association of sedentary behaviour with metabolic syndrome: A meta-analysis. PLoS ONE.

[16] Tremblay M.S., LeBlanc A.G., Kho M.E., Saunders T.J., Larouche R., Colley R.C., Goldfield G., Connor Gorber S. (2011). Systematic review of sedentary behaviour and health indicators in school-aged children and youth. Int. J. Behav. Nutr. Phys. Act..

[17] Statistical Bureau of Sichuan, NBS Survey Office in Sichuan (2012). Sichuan Statistical Yearbook.

[18] Cheng G., Duan R., Kranz S., Libuda L., Zhang L. (2016). Development of a dietary index to assess overall diet quality for Chinese school-aged children: The Chinese children dietary index (CCDI). J. Acad. Nutr. Diet..

[19] Schofield W.N. (1985). Predicting basal metabolic rate, new standards and review of previous work. Hum. Nutr. Clin. Nutr..

[20] Huang Y.J., Wong S.H., Salmon J. (2009). Reliability and validity of the modified chinese version of the children’s leisure activities study survey (class) questionnaire in assessing physical activity among Hongkong children. Pediatr. Exerc. Sci..

[21] Matthews C.E., Shu X.O., Yang G., Jin F., Ainsworth B.E., Liu D., Gao Y.T., Zheng W. (2003). Reproducibility and validity of the shanghai women’s health study physical activity questionnaire. Am. J. Epidemiol..

[22] Ainsworth B.E., Haskell W.L., Leon A.S., Jacobs D.R., Montoye H.J., Sallis J.F., Paffenbarger R.S. (1993). Compendium of physical activities: Classification of energy costs of human physical activities. Med. Sci. Sports Exerc..

[23] Ridley K., Ainsworth B.E., Olds T.S. (2008). Development of a compendium of energy expenditures for youth. Int. J. Behav. Nutr. Phys. Act..

[24] Department of Health and Human Services: 2008 Physical Activity Guidelines for Americans. http://www.health.gov/paguidelines/pdf/paguide.pdf.

[25] Chinese Students Constitution and Health Research Group (2010). Reports on the Physical Fitness and Health Research of Chinese School Students.

[26] Slaughter M.H., Lohman T.G., Boileau R.A., Horswill C.A., Stillman R.J., Van Loan M.D., Bemben D.A. (1988). Skinfold equations for estimation of body fatness in children and youth. Hum. Biol..

[27] VanItallie T.B., Yang M.U., Heymsfield S.B., Funk R.C., Boileau R.A. (1990). Height-normalized indices of the body’s fat-free mass and fat mass: Potentially useful indicators of nutritional status. Am. J. Clin. Nutr..

[28] Ji C.Y., Working Group on Obesity in China (2005). Report on childhood obesity in China (1)—Body mass index reference for screening overweight and obesity in Chinese school-age children. Biomed. Environ. Sci. BES.

[29] Li H., Ji C.Y., Zong X.N., Zhang Y.Q. (2009). Body mass index growth curves for Chinese children and adolescents aged 0 to 18 years. Zhonghua Er Ke Za Zhi Chin. J. Pediatr..

[30] Burrows T.L., Martin R.J., Collins C.E. (2010). A systematic review of the validity of dietary assessment methods in children when compared with the method of doubly labeled water. J. Am. Diet. Assoc..

[31] Yang Y.X., Wang G.Y., Pan X.-C. (2004). China Food Composition.

[32] Tanner J.M. (1986). Normal growth and techniques of growth assessment. Clin. Endocrinol. Metab..

[33] Chen C., Lu F.C. (2004). The guidelines for prevention and control of overweight and obesity in Chinese adults. Biomed. Environ. Sci. BES.

[34] Gorely T., Biddle S.J., Marshall S.J., Cameron N. (2009). The prevalence of leisure time sedentary behaviour and physical activity in adolescent boys: An ecological momentary assessment approach. Int. J. Pediatr. Obes..

[35] Kann L., Kinchen S., Shanklin S.L., Flint K.H., Kawkins J., Harris W.A., Lowry R., Olsen E.O., McManus T., Chyen D. (2014). Youth risk behavior surveillance—United States, 2013. MMWR. Surveill. Summ..

[36] Scully M., Dixon H., White V., Beckmann K. (2007). Dietary, physical activity and sedentary behaviour among Australian secondary students in 2005. Health Promot. Int..

[37] Leatherdale S.T., Wong S.L. (2008). Modifiable characteristics associated with sedentary behaviours among youth. Int. J. Pediatr. Obes..

[38] Cliff D.P., Okely A.D., Burrows T.L., Jones R.A., Morgan P.J., Collins C.E., Baur L.A. (2013). Objectively measured sedentary behavior, physical activity, and plasma lipids in overweight and obese children. Obesity.

[39] Brunner E.J., Chandola T., Marmot M.G. (2007). Prospective effect of job strain on general and central obesity in the Whitehall ii study. Am. J. Epidemiol..

[40] Pretorius K., Van Niekerk A. (2015). Childhood psychosocial development and fatal injuries in Gauteng, South Africa. Child Care Health Dev..

[41] Eisenmann J.C., Heelan K.A., Welk G.J. (2004). Assessing body composition among 3- to 8-year-old children: Anthropometry, bia, and dxa. Obes. Res..

